# Nutrient Transitions Are a Source of Persisters in *Escherichia coli* Biofilms

**DOI:** 10.1371/journal.pone.0093110

**Published:** 2014-03-25

**Authors:** Stephanie M. Amato, Mark P. Brynildsen

**Affiliations:** Department of Chemical and Biological Engineering, Princeton University, Princeton, New Jersey, United States of America; Institut Pasteur, France

## Abstract

Chronic and recurrent infections have been attributed to persisters in biofilms, and despite this importance, the mechanisms of persister formation in biofilms remain unclear. The plethora of biofilm characteristics that could give rise to persisters, including slower growth, quorum signaling, oxidative stress, and nutrient heterogeneity, have complicated efforts to delineate formation pathways that generate persisters during biofilm development. Here we sought to specifically determine whether nutrient transitions, which are a common metabolic stress encountered within surface-attached communities, stimulate persister formation in biofilms and if so, to then identify the pathway. To accomplish this, we established an experimental methodology where nutrient availability to biofilm cells could be controlled exogenously, and then used that method to discover that diauxic carbon source transitions stimulated persister formation in *Escherichia coli* biofilms. Previously, we found that carbon source transitions stimulate persister formation in planktonic *E. coli* cultures, through a pathway that involved ppGpp and nucleoid-associated proteins, and therefore, tested the functionality of that pathway in biofilms. Biofilm persister formation was also found to be dependent on ppGpp and nucleoid-associated proteins, but the importance of specific proteins and enzymes between biofilm and planktonic lifestyles was significantly different. Data presented here support the increasingly appreciated role of ppGpp as a central mediator of bacterial persistence and demonstrate that nutrient transitions can be a source of persisters in biofilms.

## Introduction

Bacterial persisters are rare, phenotypic variants, whose hallmark characteristic is a transient, yet extraordinary, ability to tolerate antibiotics while their surrounding kin are killed. [1] Biofilms contain persisters, and this phenotypic state has been hypothesized to underlie why biofilm infections often relapse. [2], [3] Persisters form during biofilm growth, and despite the identification of several important mediators, including ppGpp,[4]–[6] Lon, [4] RecA, [6] and YafQ, [7] the aspects of biofilm development that generate persisters, along with their respective pathways, remain ill-defined. The biofilm life-style includes numerous qualities conducive to persister formation, including slower growth, [8], [9] decreased metabolism, [10] quorum signaling, [11], [12] oxidative stress, [13], [14] and nutrient heterogeneity. [15], [16] This suggests that the composition of persister sub-populations in mature biofilms is likely heterogeneous,[10], [17]–[19] consisting of persisters formed from different pathways in response to various signals throughout biofilm growth.

Recently, we identified a persister formation pathway in response to nutrient transitions in planktonic *E. coli*. [16] Nutrient transitions are abundant in biofilms, as cells at the periphery consume favorable substrates and leave less favorable substrates and waste products available to cells deeper in the film. [5], [20] Together, these phenomena suggest that nutrient transitions may be a source of persisters in biofilms. However, several studies have found that genes important to persistence in one lifestyle, biofilm or planktonic, are dispensable to persistence in the other. [6], [7] These observations highlight the necessity to test the functionality of persister formation pathways identified under planktonic conditions in biofilm environments. To date, persister formation cascades, from source of stress to antibiotic tolerance, have mainly been studied in planktonic systems, [12], [14], [16], [21] and the extent to which these pathways operate in biofilms remains an open question. Conversely, specific genes important for biofilm persistence have been identified [4]–[7], but their placement in formation cascades are just beginning to be elucidated.[4]–[6].

Here we sought to determine whether carbon source transitions within biofilms generate persisters, and if so, to then identify the underlying pathway. To accomplish this, we established a biofilm culturing method where nutrient availability to cells could be controlled exogenously. Using this method, we found that diauxic transitions stimulate persister formation in *E. coli* biofilms through a pathway that involves the ppGpp synthase, RelA, and nucleoid-associated proteins (NAPs), FIS and HU. [22], [23] This pathway is qualitatively similar to the one found in planktonic cultures, with the exceptions of the removal of one ppGpp synthase to alter stringent control was sufficient to eliminate persister formation and only a subset of the NAPs involved in persister formation in planktonic conditions were found to participate in persister formation in biofilms. These findings provide a more thorough understanding of the importance of ppGpp to persistence in biofilms and point to nutrient transitions as an inherent characteristic of biofilm growth that has the capacity for persister generation.

## Materials and Methods

### Bacterial Strains, Plasmids, and Biofilm Growth Conditions

*E. coli* MG1655 was the wild-type strain used in this study. Its genetic mutants and plasmids used in this study are displayed in Table S3. Primers used to construct plasmids are displayed in Table S4. Separate colonies were used for each of three replicate experiments. Biofilm experiments were performed using colony biofilms. [20] For these experiments, cells from −80°C stock were grown for 4 h in LB, diluted 1∶100 into 2 mL of 10 mM glucose M9 media, and grown overnight for 16 h at 37°C and 250 rpm. The overnight culture was diluted into fresh M9 media containing 15 mM carbon content to an optical density at 600 nm (OD_600_) of 0.01 and 100 μL aliquots were inoculated onto sterile, polyethersulfone (PES) membranes (0.2 μm pore size, 25 mm diameter, Pall Corporation, Ann Arbor, MI) positioned on M9 minimal agar plates containing either 60 mM carbon content of carbon source, no carbon, or LB. Plates were incubated at 37°C. To monitor growth, PES membranes were aseptically removed from the agar, vortexed in 2 mL of sterile PBS for 1 minute, and the OD_600_ of the resulting cell suspensions were measured. Growth was reported as a fold change in OD_600_ (FC_OD600_)_,_ which is the ratio of cells present on the membrane to the cells inoculated onto the membrane.

### Carbon Source Transition Assay

**Figure 1:**
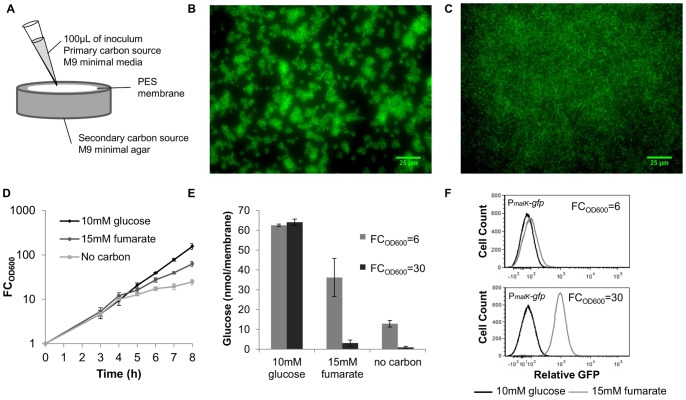
Experimental approach to control carbon source transitions in biofilms. (A) A schematic of our experimental setup where cells and the primary carbon source are applied to a PES membrane set atop agar containing the secondary carbon source, glucose, or no carbon. (B) Biofilms expressing GFP were grown to FC_OD600_ = 30. Membranes were aseptically removed from the agar and analyzed using fluorescence microscopy. (C) Cells expressing GFP were inoculated into 25 mL of 10 mM glucose at 0.01 OD_600_ and after ∼5 doublings, ∼10^7^ CFU were inoculated onto a sterilized PES membrane and analyzed using fluorescence microscopy. (D) PES membranes atop agar containing 10 mM glucose, 15 mM fumarate, and no carbon were inoculated with wild-type cells at 0.01 OD_600_ in 2.5 mM glucose and incubated at 37°C. The OD_600_ was measured at specified time intervals and FC_OD600_ was determined. One exponential growth phase was observed for glucose samples. Two regimes of exponential growth were observed for glucose-fumarate samples and no carbon sample exhibited limited growth after glucose exhaustion. (E) Glucose concentration measurements were taken at each persister sampling (FC_OD600_ = 6 and FC_OD600_ = 30) for glucose and glucose-fumarate samples and at FC_OD600_ = 6 and 2 h post glucose exhaustion for the no carbon sample. (F) P*_malK_*-*gfp* GFP distribution at FC_OD600_ = 6 and FC_OD600_ = 30 in glucose-fumarate and glucose samples. Data are averages of ≥3 independent experiments for (D) and (E) and data are representative samples of 3 independent experiments for (B), (C), and (F) and error bars indicate standard deviation.

Colony biofilms were grown as described by diluting the overnight into fresh 15 mM carbon content media, inoculating onto PES membranes atop the desired agar plates, and incubating at 37°C. At desired time points, membranes containing colony biofilms were aseptically removed from the agar, vortexed in 2 mL of PBS for 1 minute, and the OD_600_ was measured. OD_600_ was monitored for over 8 h of growth with specified secondary carbon sources and no carbon. Persister measurements were taken prior to glucose exhaustion (FC_OD600_∼14, Figure 1) at FC_OD600_ = 6 and after glucose exhaustion at FC_OD600_ = 30.

### Microscopy of Colony Biofilms

*E. coli* MG1655 was modified to contain a chromosomally integrated *lacI^q^* promoter in place of the *lacI* promoter [10] and a chromosomally integrated P_T5_ under the control of two lac operator sites [24] followed by *gfp* in place of *lacZYA* to generate the SA034 strain, which was used to image cells grown on the PES membranes. Cells from −80°C stock were grown for 4 h in LB, diluted 1∶100 into 2 mL of 10 mM glucose M9 media with 2 mM isopropyl β-D-1-thiogalactopyranoside (IPTG), and grown overnight for 16 h at 37°C and 250 rpm.

For biofilm samples, the overnight culture was diluted into fresh 2.5 mM glucose M9 media with 2 mM IPTG to an OD_600_ of 0.01 and 100 μL aliquots were inoculated onto sterile, PES membranes positioned on 10 mM glucose M9 minimal agar plates containing 2 mM IPTG. Plates were incubated at 37°C to FC_OD600_ = 30 (∼5 doublings). For planktonic samples, the overnight culture was diluted in 25 mL of fresh 10 mM glucose M9 media with 2 mM IPTG to an OD_600_ of 0.01. Cells were grown at 37°C and 250 rpm until ∼5 doublings and then ∼10^7^ cells (approximately the number of cells present on the biofilm at FC_OD600_ = 30) were applied to a sterile PES membrane.

Membranes were immobilized on a glass slide and a cover slip was placed over them prior to imaging. Imaging was performed using a Nikon Ti-E microscope (Nikon, Melville, NY), a 20X Plan Fluor Nikon objective (0.45 NA), a Chroma 89014 filter set (Chroma, Bellows Falls, VT) with an ET490/20x excitation filter and an ET535/50 m emission filter, a Prior Lumen 200 Pro fluorescence illuminator, and an Andor Clara camera.

### Transcriptional Reporters

MG1655 possessing pSA03 [16] was used as a cAMP transcriptional reporter as indicated. Kanamycin (50 μg/mL) was present during growth for plasmid retention. Cells were prepared as described above. At FC_OD600_ = 6 (before glucose exhaustion) and at FC_OD600_ = 30 (after glucose exhaustion), membranes were aseptically removed from the agar and vortexed in 2 mL of PBS.

All strains including controls were analyzed by LSRII (BD Biosciences, San Jose, CA) flow cytometer. Microorganisms were determined using forward and side scatter parameters (FSC and SSC) and MG1655 carrying pUA66 as a control. The bacteria were assayed with a laser emitting at 488 nm for GFP, and green fluorescence was collected by 525/50 bandpass filter. Data were acquired and analyzed using FACSDiVa software (BD Biosciences, San Jose, CA).

### Glucose Measurements

Cells were prepared as described, and at FC_OD600_ = 6 (before glucose exhaustion) and FC_OD600_ = 30 (after glucose exhaustion), membranes were aseptically removed from the agar and vortexed in 1 mL of PBS. For the no carbon control, samples were taken at FC_OD600_ = 6 and 2 h post-glucose exhaustion. Glucose was quantified using the Amplex Red Glucose/Glucose Oxidase Kit (Invitrogen, Eugene, OR).

### Persistence Measurements

Persisters were enumerated by determining the number of colony forming units (CFU) after exposure to 10 μg/mL ofloxacin or 750 μg/mL ampicillin for 5 h. Five hours was sufficient to provide CFU measurements within the second phase of a biphasic, time-dependent kill curve, which is required for persister measurements.[25]–[27] The antibiotic concentrations used were selected from those that lie on the second phase of a concentration-dependent kill curve (Figure S1), and thus were able to provide concentration-independent persister measurements.[25]–[27] At specified FC_OD600_, colony biofilms on membranes were treated with 200 μL of antibiotic solution applied to the top of the membrane and incubated at 37°C. At designated time points post antibiotic treatment, membranes were aseptically removed from the agar, vortexed in 2 mL of PBS, washed and serially diluted in PBS, and 10 μL was spotted onto LB agar. For ampicillin-treated cells, the whole sample was plated on LB agar to increase the limit of detection. Cells in PBS were stored at 4°C prior to plating as necessary, and it was confirmed that such storage did not affect CFU measurements when compared to samples plated immediately. Plates were incubated for 16 h at 37°C and CFU were measured to determine persister counts. 10–100 colonies were counted for each data point. [28].

### Statistical Analysis

Statistical significance was assessed using 2-tailed t-tests with unequal variances. Persister data obtained from the 5 h post antibiotic treatment CFU measurements were statistically analyzed. We previously confirmed that the data from persister assays, CFU measurements after 5 h antibiotic treatment, can be treated as near-normally distributed with a larger sample dataset. [16] The threshold for significance was set to p-values <0.05.

## Results and Discussion

### Establishment of a Method to Exogenously Control Carbon Source Availability in Colony Biofilms

Bacteria can exhibit either diauxic or non-diauxic growth when grown in the presence of two carbon sources. [29] During diauxic growth, the preferential carbon source is consumed in the first growth phase, whereas the less favorable secondary carbon source supports growth during the second growth phase. The two growth phases are separated by a lag period associated with physiological changes required for growth on the secondary carbon source. [30] Non-diauxic growth can exhibit preferential carbon source consumption, but lacks the intermediate lag period. [31] We have previously demonstrated that diauxic carbon source transitions stimulate persister formation in planktonic *E. coli* cultures. [16] Here, we sought to investigate whether carbon source transitions stimulate persister formation during biofilm growth, and if so, identify the formation pathway responsible.

To study persister formation from carbon source transitions in biofilms, we required an experimental system where only biofilm cells were present and nutrient availability could be controlled exogenously. Biofilms are often grown in the presence of planktonic cells, [32], [33] but results from these systems are complicated by biofilm dispersal, and when considering rare events such as persisters, uncertainty arises as to whether a cell originated from the biofilm or planktonic sub-population. Given these considerations, we used the colony biofilm culturing method, where cells are grown on nutrient-permeable membranes positioned atop agar plates. [5], [20], [34] In this method, which has been used in previous persistence studies, [4], [5] all bacteria are surface-attached biofilm cells and planktonic cells are absent. This culturing method also allowed exogenous control of nutrient availability to biofilm cells (Figure 1A). Glucose, the primary carbon source, was delivered with cells to the top of PES membranes and secondary carbon sources or controls (glucose and no carbon) were provided in the agar. Young biofilms were investigated to avoid the in-film nutrient gradients that are present in mature colony biofilms, which would complicate the analysis of a single carbon source transition and its role in persister formation. [15], [20] To demonstrate that the colony biofilm method produced young biofilms on the timescale of our experiments, we imaged colony biofilms at FC_OD600_ = 30 using fluorescence microscopy and a strain producing GFP (Figure 1B and 1C). The presence of cell clusters in the biofilm sample compared to a sample where an equal number of planktonic cells were seeded onto the membrane just prior to imaging demonstrates that these young films are immobilized cells growing in surface-attached communities.

To demonstrate that exogenous control of nutrient availability was achieved with the colony biofilm method, we monitored growth, quantified the glucose concentration in membranes, and utilized a transcriptional reporter of glucose exhaustion. Using 2.5 mM glucose in the inoculums, similar growth for films grown on agar containing glucose, fumarate, and no carbon was observed, indicating a common period of glucose consumption (Figure 1D). Fumarate was used here as a representative diauxic carbon source, and analogous measurements with additional secondary carbon sources are presented in the Supporting Information (Figure S2). At FC_OD600_∼14, growth rates of the fumarate and no carbon samples decreased, suggesting a transition away from glucose-replete conditions. Residual growth in the no carbon sample was due to trace glucose levels, whereas growth in the fumarate sample, which far exceeded that of the no carbon sample, signified fumarate catabolism. This was confirmed by quantifying glucose in the membranes (Figure 1E). Since glucose exhaustion triggers the production of cAMP [35] resulting in cAMP-CRP transcriptional activity, we used a cAMP-CRP transcriptional reporter [16] to demonstrate that cAMP-CRP is comparable between glucose and fumarate samples during the common period of glucose consumption, and cAMP-CRP activity increased in the fumarate sample after glucose depletion (Figure 1F). These flow cytometry data, which consist of only a single, distinct peak for each sample, also suggest that the biofilm cells were exposed to similar nutrient environments. Together these data demonstrate that our experimental setup was successful in achieving well-controlled carbon source transitions in biofilms.

### Carbon Source Transitions Stimulate Persister Formation

**Figure 2:**
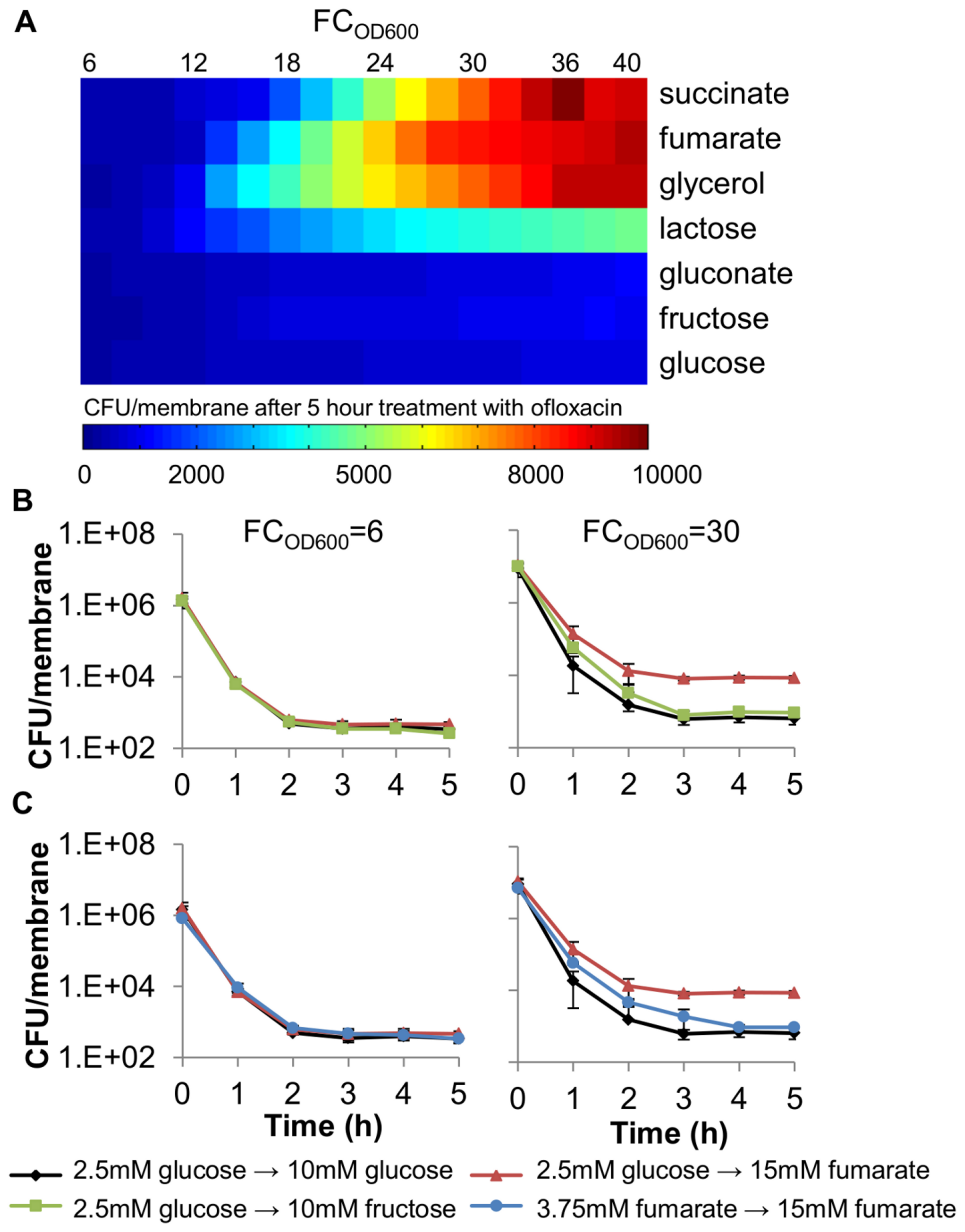
Diauxic shifts stimulate persister formation in biofilms. (A) *E. coli* were grown on glucose as the primary carbon source and a panel of secondary carbon sources. At hourly time points, biofilms were challenged with 200 μl of 10 μg/mL ofloxacin for 5 h, aseptically removed from the agar, vortexed in 2 mL PBS for 1 minute, washed, and plated to measure CFUs. To construct the color plot as a function of FC_OD600_, as needed values plotted were interpolated from two adjacent measurements. Raw values are presented in Table S2. (B) Diauxic growth (glucose-fumarate) results in significant persister formation (p<0.05), whereas non-diauxic growth does not (p>0.05) (glucose and glucose-fructose) when comparing persister levels 5 h post-antibiotic treatment. Time on the x-axis represents time after antibiotic treatment. (C) Growth on fumarate is not responsible for persister formation in glucose-fumarate samples, as evidence by sole fumarate control, which contained fumarate as the only carbon source both in the inoculum and agar. Data are averages of ≥3 independent experiments, error bars indicate standard deviation, and significance was assessed using the null hypothesis that the mean CFU levels in two sample sets were equal.

After establishing a functional system for analyzing carbon source transitions in biofilms, we examined if transitions stimulate persister formation. *E. coli* persister levels were measured using ofloxacin throughout growth using glucose as the primary carbon source and a panel of secondary carbon sources that are diauxic or non-diauxic with glucose. The antibiotic treatment was delivered to the biofilms as 200 μL of 10 μg/mL ofloxacin evenly distributed atop the membrane to ensure full treatment of the biofilm. It was observed that diauxic media exhibited an increase in persisters to ofloxacin upon glucose exhaustion compared to the sole glucose control (Figure 2A). The non-diauxic secondary carbon sources, fructose [36] and gluconate [37] did not exhibit an increase in persisters to ofloxacin after glucose exhaustion. These results suggested that diauxic transitions stimulated formation of persisters to ofloxacin in biofilms. In addition, we tested whether carbon source transitions stimulate formation of persisters to additional antibiotics. When the aminoglycoside gentamicin was tested we observed that all cells were killed (Figure S1), a result that was expected given that glucose potentiates aminoglycoside activity in persisters. [38], [39] However, we found that carbon transitions also stimulated formation of persisters to the β-lactam ampicillin. Using glucose as the primary carbon source and a panel of secondary carbon sources that are diauxic or non-diauxic with glucose and treating with 200 μL of 750 μg/mL ampicillin, we observed no persister formation prior to the transition at FC_OD600_ = 6, but observed persister formation for fumarate and succinate after the transition at FC_OD600_ = 30 (Figure S3A). These results demonstrate that persister formation in biofilms in response to carbon source transitions is not specific to fluoroquinolones. In addition, these data suggest the existence of distinct pathways, since different sets of secondary carbon sources stimulate ofloxacin and ampicillin persister formation (Figure S3B).

To identify the underlying formation pathway for ofloxacin persisters, we focused on glucose-fumarate transitions, since they elicited the strongest persister formation response and were previously studied in planktonic culture. [16] For comparison, glucose-fructose transitions, which are non-diauxic, were investigated. Persister measurements were taken during growth on glucose (FC_OD600_ = 6) and after glucose exhaustion (FC_OD600_ = 30). During exponential growth on glucose, all persister levels were the same independent of the secondary carbon source present (Figure 2B). Persister measurements during exponential growth on the secondary carbon source exhibited a statistically significant 13-fold increase in persisters for the glucose-fumarate samples and an insignificant 1.4-fold increase for the glucose-fructose samples when compared to persister levels in the glucose-only samples at the same FC_OD600_.

To establish that the increase in persisters was due to the transition from glucose to fumarate and not from the slower growth on fumarate, persister measurements were taken at the same film densities during growth on fumarate as the sole carbon source (fumarate provided in the inoculum and agar) (Figure 2C). The persister levels for the culture grown in the fumarate-only samples were 1.4-fold higher and not statistically different from persister levels for glucose-only samples, but were 9-fold lower and statistically different from those for the glucose-fumarate samples, which suggests that the increase in persisters was a result of the carbon source transition and not slower growth on fumarate.

### Persisters Form through a RelA-dependent Mechanism

We next sought to determine the molecular mechanism responsible for persister formation from carbon source transitions in biofilms. Upon glucose limitation, the stringent response is activated and the metabolites pppGpp and ppGpp, collectively termed ppGpp for subsequent discussion, are synthesized by both RelA and SpoT. [40], [41] RelA is a ribosome-associated ppGpp synthase, whereas SpoT has both synthase and hydrolase activity. [41] We have shown previously that persister formation from carbon source transitions in planktonic cultures is dependent on the ppGpp biochemical network. [16] Therefore, we tested whether ppGpp is also essential for persister formation due to carbon source transitions in biofilms.

**Figure 3:**
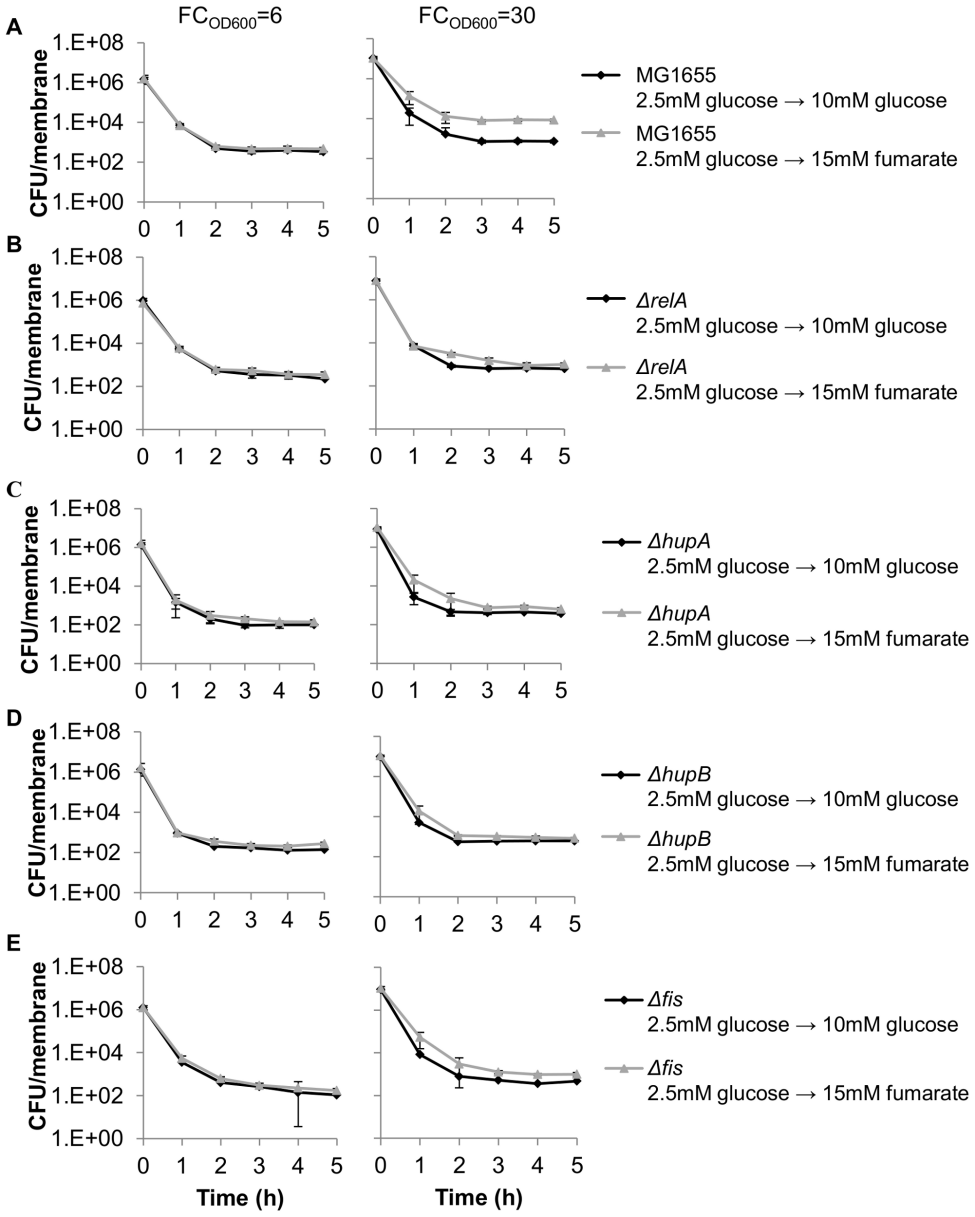
Genes required for persister formation from carbon source transitions in biofilms. Cells were challenged with 200 μL of 10 μg/mL ofloxacin at FC_OD600_ = 6 and FC_OD600_ = 30, representing growth on glucose and growth after glucose exhaustion, respectively (except for glucose-only sample). Carbon source transitions resulted in significant persister formation for (A) wild-type. (B) *ΔrelA* eliminated persister formation compared to wild-type (p<0.05). Components of 2 NAPs (C) *ΔhupA,* (D) *ΔhupB,* and (E) *Δfis* eliminated persister formation compared to wild-type (p<0.05). Time on the x-axis represents time after antibiotic treatment. Data are averages of 3 independent experiments, error bars indicate standard deviation, and significance was assessed using the null hypothesis that the mutant mean fold-change in persisters was equal to the wild-type fold-change in persisters.

We measured persisters prior to and after glucose exhaustion in *ΔrelA* (Figure 3, Table S1). Deletion of the ribosome-dependent ppGpp synthase RelA resulted in an insignificant 1.6-fold increase in persisters when comparing glucose-fumarate to glucose-only samples. The complemented *ΔrelA* strain restored the persister formation phenotype confirming that RelA is required for persister formation from carbon source transitions in biofilms (Figure S4). Interestingly, under planktonic conditions, *ΔrelA* was only found to reduce the quantity of persisters formed, whereas deletion of both ppGpp synthases (*ΔrelAΔspoT)* was required to eliminate persister formation. [16] Although the SpoT synthase has been shown to be activated in response to carbon source starvation [42], [43] and may experience increased activity during the transition, *ΔspoT* is not viable in a *relA^+^* background [44] and *ΔrelAΔspoT* is auxotrophic for several amino acids. [45] Since directly assessing the role of SpoT in the carbon source transition model would have required amino acid supplementation, and complete elimination of persister formation was achieved with *ΔrelA*, the involvement of SpoT was not further explored.

To validate that *E. coli* experiences a ppGpp-dependent stringent response during carbon source transitions in biofilms, we monitored levels of stable ribosomal RNA (rRNA) in wild-type and its *ΔrelA* derivative during the transition using quantitative PCR (qPCR). The seven rRNA operons are regulated by the stringent response, and, when levels of ppGpp are high, transcription of the rRNA is repressed. [41] However, in *ΔrelA* this inhibition does not occur and levels of stable rRNA have been shown to be ∼2- to 10-fold higher than that of a *relA^+^* strain in similar conditions.[46]–[50] Within colony biofilms, we found that both 23 S and 16 S RNA expression was ∼2-fold higher in *ΔrelA* than wild-type during carbon source transitions at FC_OD600_ = 14 (Figure S4, Table S5). These data confirm that ppGpp in *ΔrelA,* where persister formation was eliminated, was lower than in wild-type where persister formation was observed, suggesting that increased ppGpp levels are required for persister formation from carbon source transitions.

### ppGpp-dependent Persister Formation in Biofilms Requires NAPs

We next sought to determine how ppGpp in biofilms increases tolerance to ofloxacin, an antibiotic that targets DNA gyrase. [51] Previously, high levels of ppGpp have been observed to lead to relaxation of the chromosome, an indicator of reduced DNA gyrase activity. [52] Though the mechanism underlying this phenomenon remains ill-defined, it is known that DNA gyrase, topoisomerases I, III, and IV, and NAPs work together to control the degree of (−) supercoiling of the chromosome. [52], [53] Given the role of NAPs in (−) supercoiling, the knowledge that NAPs are under stringent control both directly and indirectly through a complex interdependent network of regulation,[53]–[56] and the discovery that several NAPs were involved in persister formation from carbon source transitions in planktonic cultures, we tested mutants of NAPs FIS, HNS, HU, IHF, and SeqA.[57]–[59].

**Figure 4:**
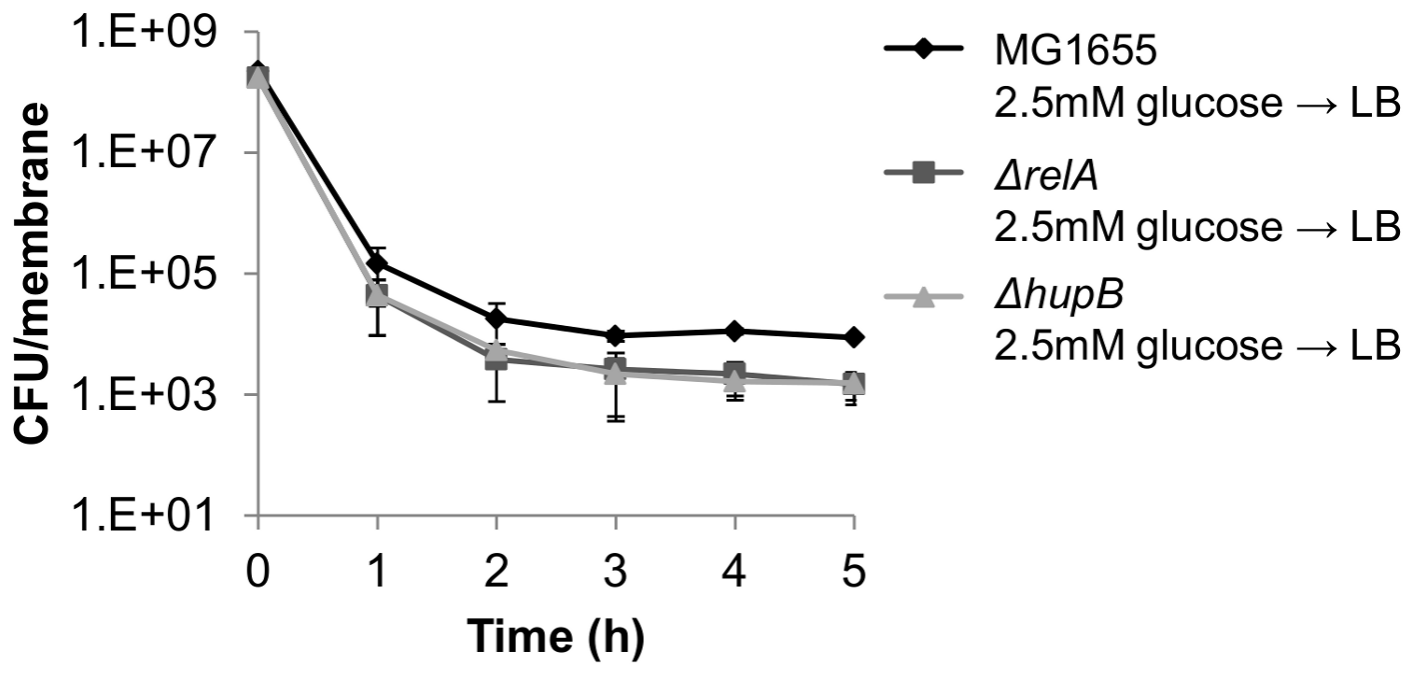
Mediators of persister formation during carbon source transitions play significant role during growth on complex media. *E. coli* in 2.5 mM glucose M9 media were inoculated onto PES membranes atop LB agar. At a FC_OD600_ = 1000, cells were challenged with 200 μL of 10 μg/mL ofloxacin. (A) MG1655 demonstrated a statistically significant 6-fold increase in persisters compared to (B) *ΔrelA,* and (C) *ΔhupB* (p-value<0.05). Time on the x-axis represents time after antibiotic treatment. Data are averages of 3 independent experiments, error bars indicate standard deviation, and significance was assessed using the null hypothesis that the mean CFU levels in two sample sets were equal.

We found that *Δfis*, *ΔhupA*, *ΔhupB,* and *ΔseqA* all removed persister formation from carbon source transitions in biofilms, whereas *ΔihfA, ΔihfB*, and *Δhns* did not eliminate persister formation (Figures 4, S5, and S6). For *Δfis*, *ΔhupA*, and *ΔhupB,* complementation with genes expressed from their native promoters on low-copy plasmids restored persister formation (Figure S7). However, *ΔseqA* could not be complemented to a significant level when expression was driven by the putative promoter 86 base pairs upstream of the SeqA start codon on the same low-copy plasmid (Figure S6). Although the possibility of additional mutations in the three Δ*seqA* colonies cannot be ruled out, the *seqA* promoter is putative and has been shown to be regulated by HU from an undefined location. [56] Given these uncertainties, complementation may be achieved with a different expression system. Interestingly, IHF was previously found to be an important mediator of persister formation in planktonic conditions, but here was not found to be a mediator in the biofilm state. Interactions of NAPs with chromosomal DNA and one another are strongly influenced by growth rate and phase, [53], [60], [61] so we reason that differences in these interactions between biofilm and planktonic lifestyles underlie why IHF was only found to be important for planktonic persister formation. Together, these results demonstrate that FIS and HU are required for persister generation in response to carbon source transitions in biofilms.

Given the direct connection between NAPs and the primary target of ofloxacin, we tested whether the same pathway was responsible for ampicillin persister formation from carbon source transitions in biofilms. When treating wild-type and *ΔhupB* with ampicillin before and after the carbon source transition, we observed no difference in persister formation between the two strains (Figure S8) suggesting that the formation pathway was specific to fluoroquinolones.

To determine whether mediators of the pathway contribute to persister formation in environments more complex than diauxic conditions, we performed analogous assays atop LB agar with mature biofilms, and found that Δ*relA* and Δ*hupB* had statistically significant fewer persisters than the wild-type, ∼6-fold (Figure 4). Although the decrease in persisters in Δ*relA* and Δ*hupB* cannot be specifically attributed to a carbon source transition, since many nutrient shifts and other stresses are present during growth of mature biofilms on complex media, [15], [20], [62] we do show that mediators identified by investigating nutrient shifts in isolation can participate more broadly in persister formation.

## Conclusions

The clinical importance of persisters has been attributed to their presence in biofilm infections. [3], [63] Despite this significance, the means by which persisters form in this bacterial life-style remain obscure. This is in part due to the environmental heterogeneity inherent to biofilm growth. Here we were able to establish an experimental protocol to study, in isolation, the effect of nutrient transitions on persistence in biofilms. Identification that diauxic carbon source transitions generate persisters to ofloxacin in biofilms led us to identify RelA and NAPs as critical mediators of the process. ppGpp has been increasingly identified as a major mediator of persistence.[4]–[6], [16], [64] Both Nguyen and colleagues and Maisonneuve and colleagues established that ppGpp was important for persister formation in mature *E. coli* biofilms, [4], [5] though the aspect of biofilm physiology that was responsible for the significance of ppGpp in persistence was undetermined. In addition, Bernier and colleagues found that ofloxacin persister formation from leucine starvation in *E. coli* biofilms was partially dependent on ppGpp. [6] Here we identified carbon source transitions as a biofilm property that generates persisters through a ppGpp-dependent mechanism. Further, we found that modulators of chromosomal (−) supercoiling were required for ppGpp to form persisters in response to carbon source transitions, providing a direct connection to the primary target of fluoroquinolones, DNA gyrase. Interestingly, Maisonneuve and colleagues found that the Lon protease was required for the ppGpp-dependent persister formation they observed. [4] We tested *Δlon* and found that removal of this protease did not eliminate persister formation in response to carbon source transitions (Figure S9). However, we note that *Δlon* did give rise to fewer persisters than wild-type, supporting its importance to persistence in *E. coli* biofilms. Increasingly, persistence has been found to depend on ppGpp, suggesting that the alarmone may be a common node for diverse formation mechanisms and thus an attractive candidate for anti-persister therapies. [65].

## Supporting Information

Figure S1 **Antibiotic killing at various concentrations.** PES membranes placed on 10 mM glucose M9 minimal media plates were inoculated with 100 μl of overnight *E. coli* MG1655 culture that had been diluted to an 0.01 OD_600_ in 2.5 mM glucose. After 6 hours of incubation at 37°C, 200 μl of (A) ofloxacin, (B) ampicillin, and (C) gentamicin solution at the indicated concentrations were placed on the membranes. Biofilms were treated with antibiotic for 5 h, separated from membranes by vortexing in PBS, washed with PBS, and plated on LB agar to measure CFUs.
(TIF)

Figure S2 **Growth of colony biofilms on glucose and a panel of secondary carbon sources.** PES membranes atop agar containing specified secondary carbon sources were inoculated with wild-type cells at 0.01 OD_600_ in 2.5 mM glucose and incubated at 37°C. The OD_600_ was measured at specified time intervals and FC_OD600_ was determined. Data are averages of ≥3 independent experiments and error bars indicate standard deviation.
(TIF)

Figure S3 **Ampicillin persister formation during carbon source transition.** PES membranes placed atop agar containing the specified secondary carbon sources were inoculated with 100 μl of overnight *E. coli* MG1655 culture that had been diluted to 0.01 OD_600_ in 2.5 mM glucose. (A) Cells were challenged with 200 μL of 750 μg/mL ampicillin at FC_OD600_ = 6 and FC_OD600_ = 30, treated for 5 h with antibiotic, aseptically removed from the agar, washed in PBS, and plated on LB agar to measure CFUs. (B) The ratio persisters enumerated after 5 h of antibiotic treatment on the specified secondary carbon to sole glucose at the noted FC_OD600_ is compared between ofloxacin and ampicillin treated films at FC_OD600_ = 6 and FC_OD600_ = 30. The persister formation after ampicillin treatment is distinct from that after ofloxacin treatment.
(TIF)

Figure S4 **RelA and the stringent response are important for persister formation in biofilms.** Complementation of RelA was carried out in MG1655 Δ*relA.* (A) MG1655 with the pUA66 promoterless vector showed a significant increase in persisters during the carbon source transition. (B) Δ*relA* pUA66 eliminated persister formation due to the carbon source transition, while (C) Δ*relA* pUA66-*relA* complemented strain exhibited a statistically significant increase in persisters due to carbon source transitions restoring the wild-type phenotype. Significance was assessed using the null hypothesis that the mean fold-change in persisters for the complemented strain was equal to the mean fold-change in persisters for the deletion strain carrying the pUA66 vector. (D) RNA from wild-type and Δ*relA* at the transition (FC_OD600_ = 14) was purified, converted to cDNA, and analyzed using qPCR to determine stringently controlled rRNA expression. Δ*relA* rRNA showed a statistically significant ∼2-fold higher expression than wild-type for both 16 S and 23 S. Significance was assessed using the null hypothesis that the mean fold-change of Δ*relA* expression to wild-type expression was equal to 1. Data are averages of ≥3 independent experiments and error bars indicate standard deviation.
(TIF)

Figure S5 **IHF and HNS are not involved in persister formation from carbon source transitions in biofilms.** Cells were challenged with 200 μL of 10 μg/mL ofloxacin at FC_OD600_ = 6 and FC_OD600_ = 30, representing growth on glucose and growth after glucose exhaustion, respectively (except for glucose-only sample). (A) *ΔihfA,* (B) *ΔihfB,* and (C) *Δhns* produced fold-change increases in persisters (glucose-fumarate persisters/glucose-only persisters) that were not significantly reduced compared to wild-type. Data are averages of 3 independent experiments, error bars indicate standard deviation, and significance was assessed using the null hypothesis that the mutant mean fold-change in persisters was equal to the wild-type fold-change in persisters.
(TIF)

Figure S6 **Complementation of FIS and HU.** Cells were challenged with 200 μL of 10 μg/mL ofloxacin at FC_OD600_ = 6 and FC_OD600_ = 30, representing growth on glucose and growth after glucose exhaustion, respectively (except for glucose-only sample). (A) *Δfis* pUA66 eliminated persister formation, while (B) *Δfis* pUA66-*dusB*-*fis* restored persister formation. Analogous results were obtained for (C) *ΔhupA* pUA66 compared to (D) *ΔhupA* pUA66-*hupA* and for (E) *ΔhupB* pUA66 compared to (F) *ΔhupB* pUA66-*hupB.* Data are averages of 3 independent experiments, error bars indicate standard deviation, and significance was assessed using the null hypothesis that the mean fold-change in persisters for the complemented strain was equal to the mean fold-change in persisters for the deletion strain carrying the pUA66 vector.
(TIF)

Figure S7 **Involvement of SeqA in persister formation from carbon source transitions.** Cells were challenged with 200 μL of 10 μg/mL ofloxacin at FC_OD600_ = 6 and FC_OD600_ = 30, representing growth on glucose and growth after glucose exhaustion, respectively (except for glucose-only sample). (A) *ΔseqA* eliminated persister formation compared to wild-type (p<0.05). (B) *ΔseqA* pUA66 also eliminated persister formation compared to the wild-type, but complementation of *ΔseqA* with (C) *ΔseqA* pUA66-*seqA* did not give a statistically significant increase in persisters compared to the *ΔseqA* pUA66 control. Data are averages of 3 independent experiments, error bars indicate standard deviation, and significance was assessed using the null hypothesis that the mean fold-change in persisters for the complemented strain was equal to the mean fold-change in persisters for the deletion strain carrying the pUA66 vector.
(TIF)

Figure S8 **Ampicillin persister formation from carbon source transitions does not depend on HU.** Cells were challenged with 200 μL of 750 μg/mL ampicillin at FC_OD600_ = 6 and FC_OD600_ = 30, representing growth on glucose and growth after glucose exhaustion, respectively (except for glucose-only sample). (A) Wild-type and (B) *ΔhupB* resulted in statistically significant 5.4-fold and 6.7-fold increase in persisters, respectively. Data are averages of 3 independent experiments, error bars indicate standard deviation, and significance was assessed using the null hypothesis that the mean CFU levels in two sample sets were equal.
(TIF)

Figure S9 **Involvement of Lon in persister formation from carbon source transitions in biofilms.** Cells were challenged with 200 μL of 10 μg/mL ofloxacin at FC_OD600_ = 6 and FC_OD600_ = 30, representing growth on glucose and growth after glucose exhaustion, respectively (except for glucose-only sample). *Δlon* did not eliminate persister formation due to a carbon source transition in biofilms. The limit of detection was 1 CFU/membrane. Data are averages of 3 independent experiments and error bars indicate standard deviation.
(TIF)

Table S1 **Glucose concentrations (nmol/membrane).** Glucose concentration measurements were taken at each FC_OD600_ = 6 and  = 30 for all strains in both 10 mM glucose and 15 mM fumarate samples. Measurements were made using an Amplex Red Glucose/Glucose Oxidase Kit (Invitrogen). Three replicates were conducted for each mutant and condition and error indicates standard deviation.
(DOC)

Table S2 **Raw values for** **Figure 2A** **.** FC_OD600_ prior to antibiotic treatment and persister measurements after 5 hours of treatment with 200 μL of 10 μg/mL ofloxacin were taken at the specified time points for a panel of secondary carbon sources using glucose as a primary carbon source. These data were used to linearly interpolate values to generate the heat map for Figure 2A.
(DOC)

Table S3 **Bacterial strains and plasmids.**
(DOC)

Table S4 **DNA primers for plasmid construction.**
(DOC)

Table S5 **DNA primers for qPCR.**
(DOC)

Materials and Methods S1 (DOCX)
