# Supplementary figures and images for: Nutrient Transitions Are a Source of Persisters in Escherichia coli Biofilms
Source: PLoS One. 2014 Mar 25;9(3):e93110. doi: 10.1371/journal.pone.0093110 (PMC3965526; doi:10.1371/journal.pone.0093110)

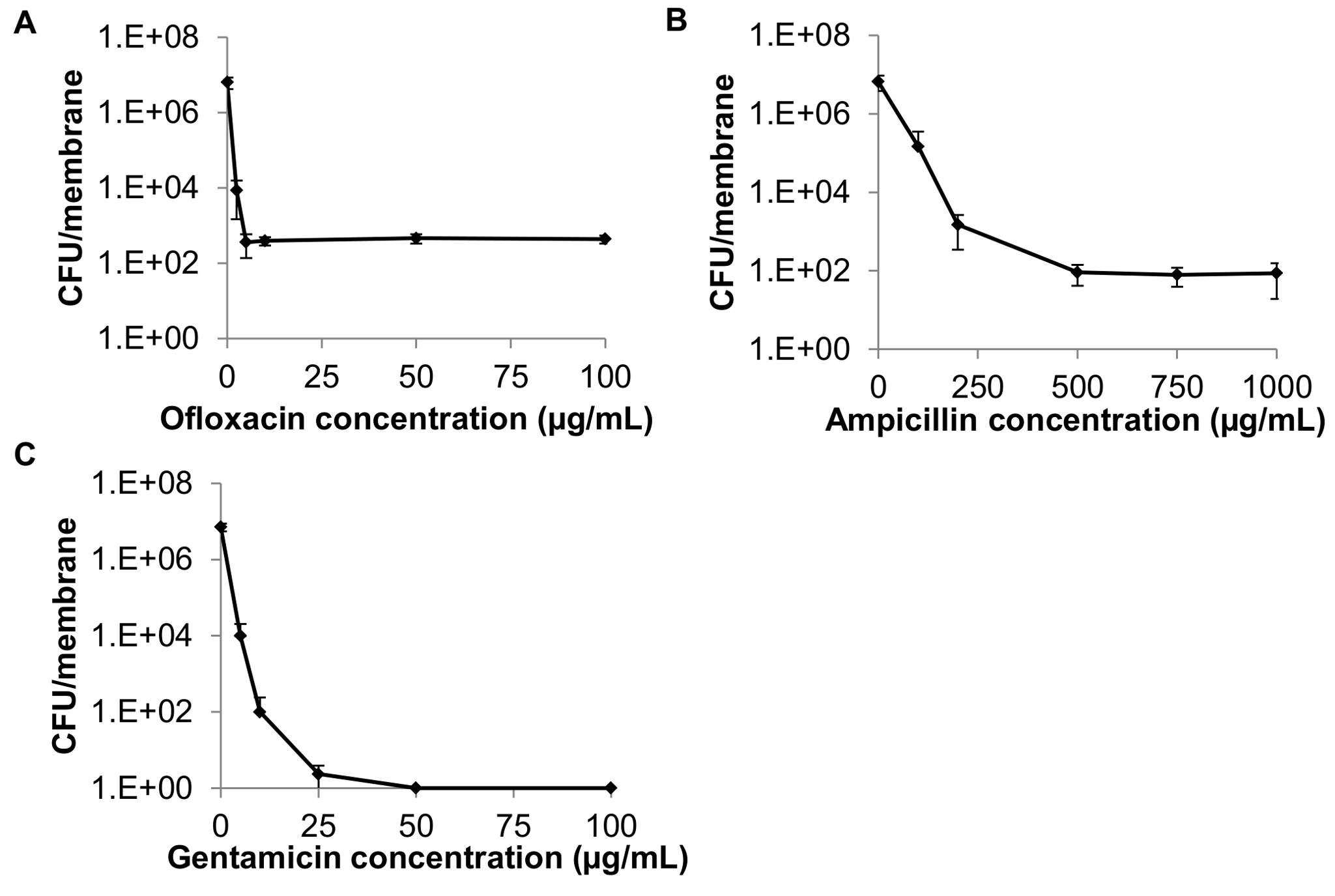

Supplement: Figure S1 — Antibiotic killing at various concentrations. PES membranes placed on 10 mM glucose M9 minimal media plates were inoculated with 100 μl of overnight E. coli MG1655 culture that had been diluted to an 0.01 OD600 in 2.5 mM glucose. After 6 hours of incubation at 37°C, 200 μl of (A) ofloxacin, (B) ampicillin, and (C) gentamicin solution at the indicated concentrations were placed on the membranes. Biofilms were treated with antibiotic for 5 h, separated from membranes by vortexing in PBS, washed with PBS, and plated on LB agar to measure CFUs. (TIF) [file pone.0093110.s001.tif]

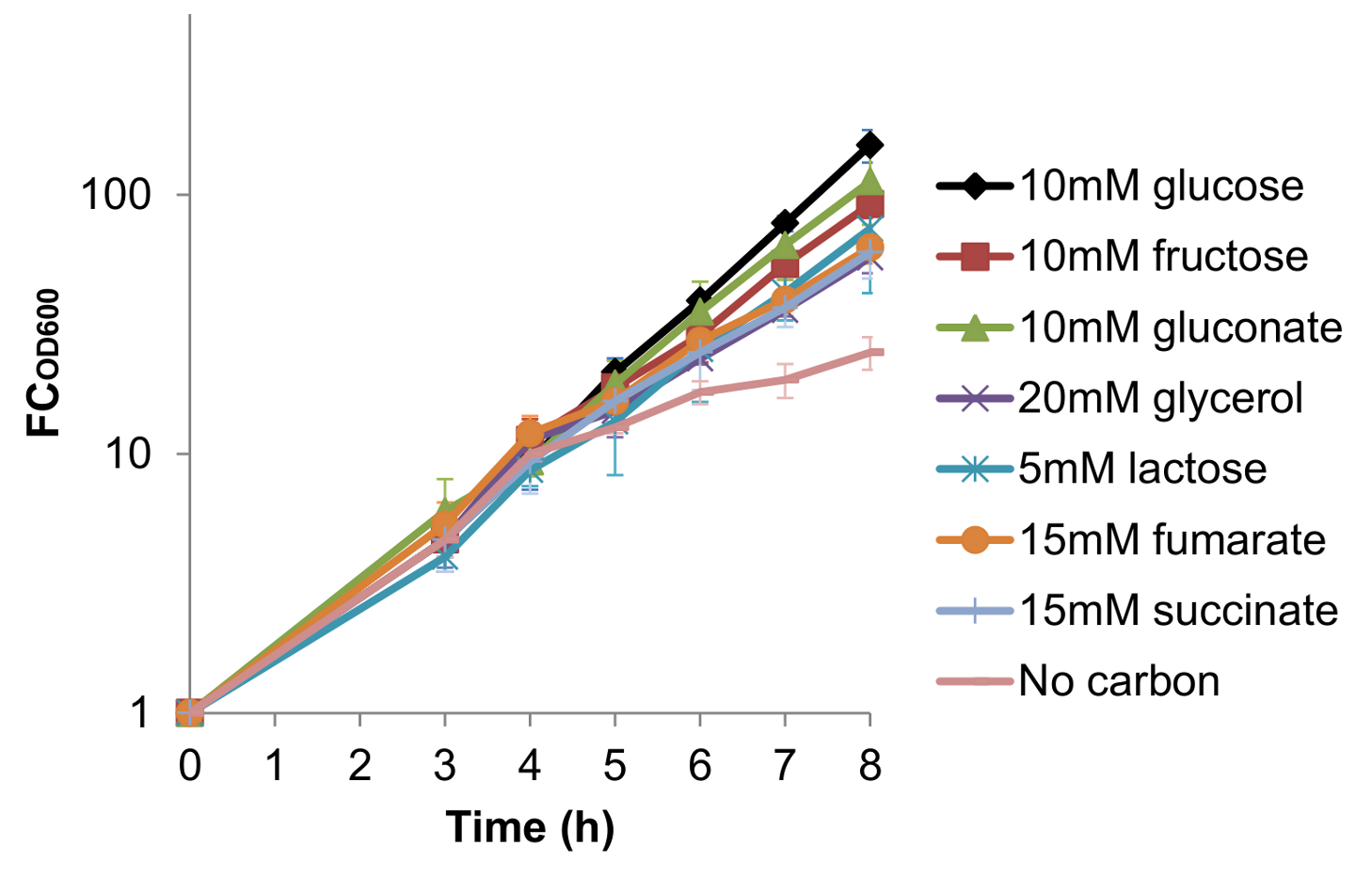

Supplement: Figure S2 — Growth of colony biofilms on glucose and a panel of secondary carbon sources. PES membranes atop agar containing specified secondary carbon sources were inoculated with wild-type cells at 0.01 OD600 in 2.5 mM glucose and incubated at 37°C. The OD600 was measured at specified time intervals and FCOD600 was determined. Data are averages of ≥3 independent experiments and error bars indicate standard deviation. (TIF) [file pone.0093110.s002.tif]

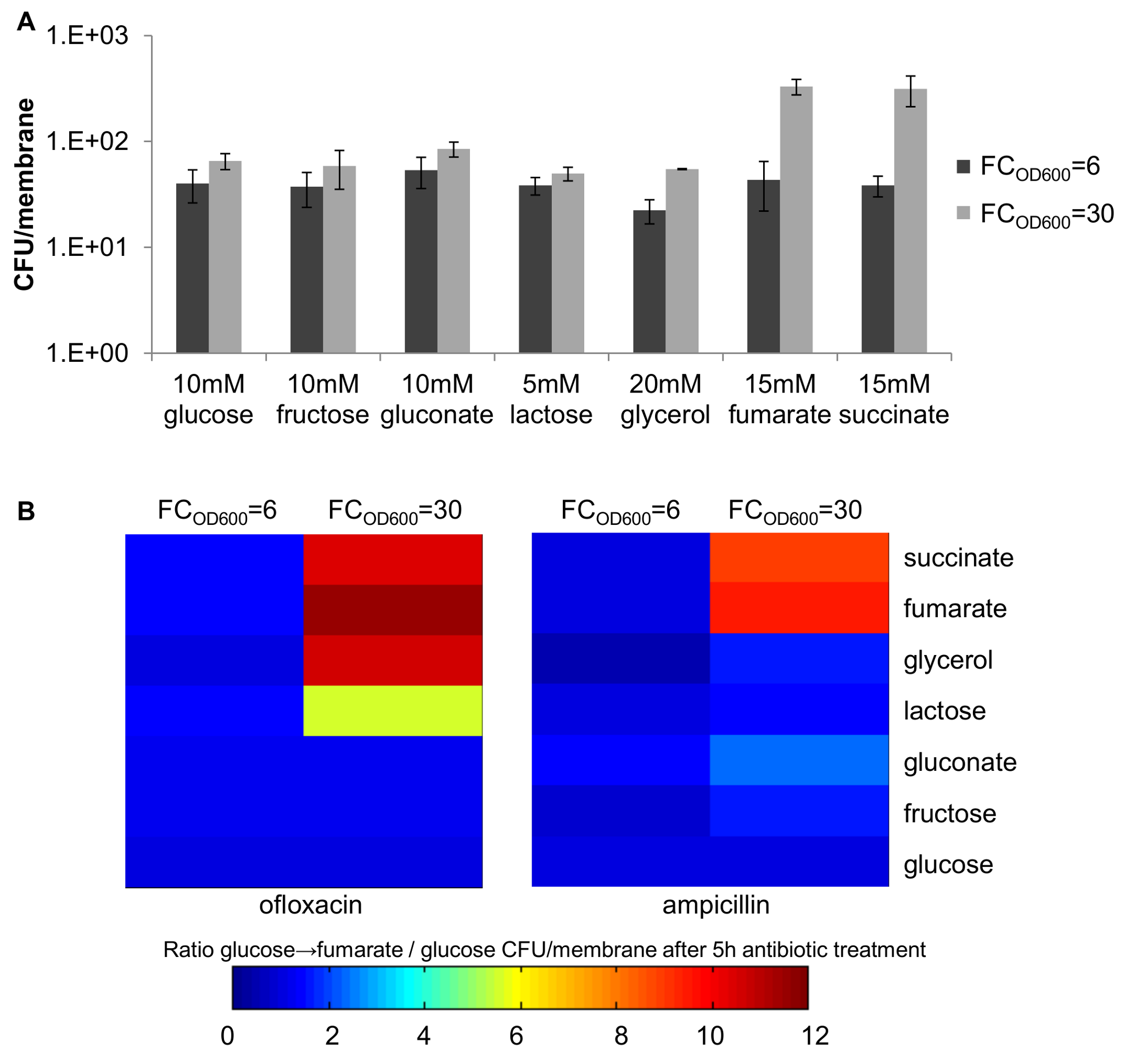

Supplement: Figure S3 — Ampicillin persister formation during carbon source transition. PES membranes placed atop agar containing the specified secondary carbon sources were inoculated with 100 μl of overnight E. coli MG1655 culture that had been diluted to 0.01 OD600 in 2.5 mM glucose. (A) Cells were challenged with 200 μL of 750 μg/mL ampicillin at FCOD600 = 6 and FCOD600 = 30, treated for 5 h with antibiotic, aseptically removed from the agar, washed in PBS, and plated on LB agar to measure CFUs. (B) The ratio persisters enumerated after 5 h of antibiotic treatment on the specified secondary carbon to sole glucose at the noted FCOD600 is compared between ofloxacin and ampicillin treated films at FCOD600 = 6 and FCOD600 = 30. The persister formation after ampicillin treatment is distinct from that after ofloxacin treatment. (TIF) [file pone.0093110.s003.tif]

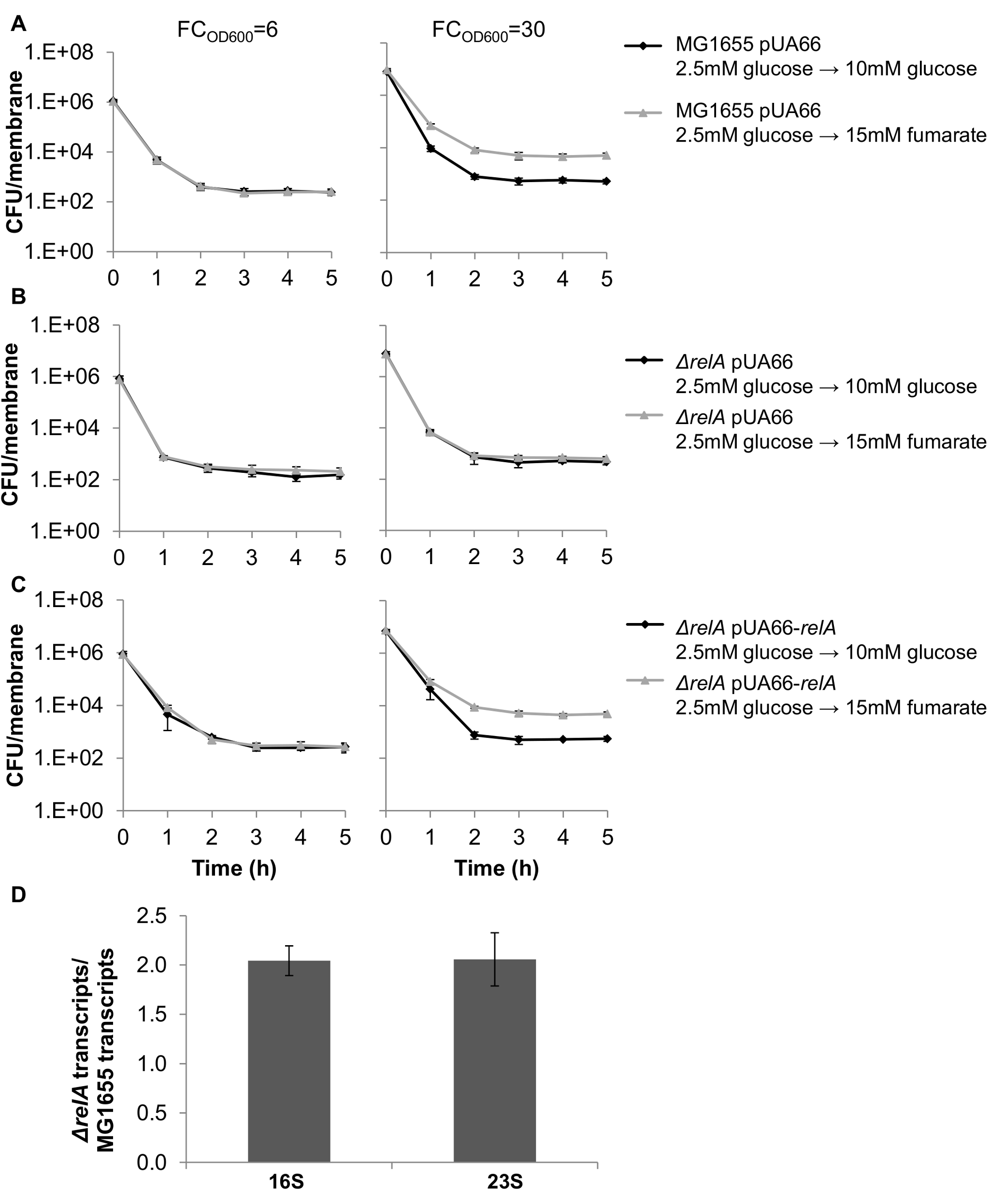

Supplement: Figure S4 — RelA and the stringent response are important for persister formation in biofilms. Complementation of RelA was carried out in MG1655 ΔrelA. (A) MG1655 with the pUA66 promoterless vector showed a significant increase in persisters during the carbon source transition. (B) ΔrelA pUA66 eliminated persister formation due to the carbon source transition, while (C) ΔrelA pUA66-relA complemented strain exhibited a statistically significant increase in persisters due to carbon source transitions restoring the wild-type phenotype. Significance was assessed using the null hypothesis that the mean fold-change in persisters for the complemented strain was equal to the mean fold-change in persisters for the deletion strain carrying the pUA66 vector. (D) RNA from wild-type and ΔrelA at the transition (FCOD600 = 14) was purified, converted to cDNA, and analyzed using qPCR to determine stringently controlled rRNA expression. ΔrelA rRNA showed a statistically significant ∼2-fold higher expression than wild-type for both 16 S and 23 S. Significance was assessed using the null hypothesis that the mean fold-change of ΔrelA expression to wild-type expression was equal to 1. Data are averages of ≥3 independent experiments and error bars indicate standard deviation. (TIF) [file pone.0093110.s004.tif]

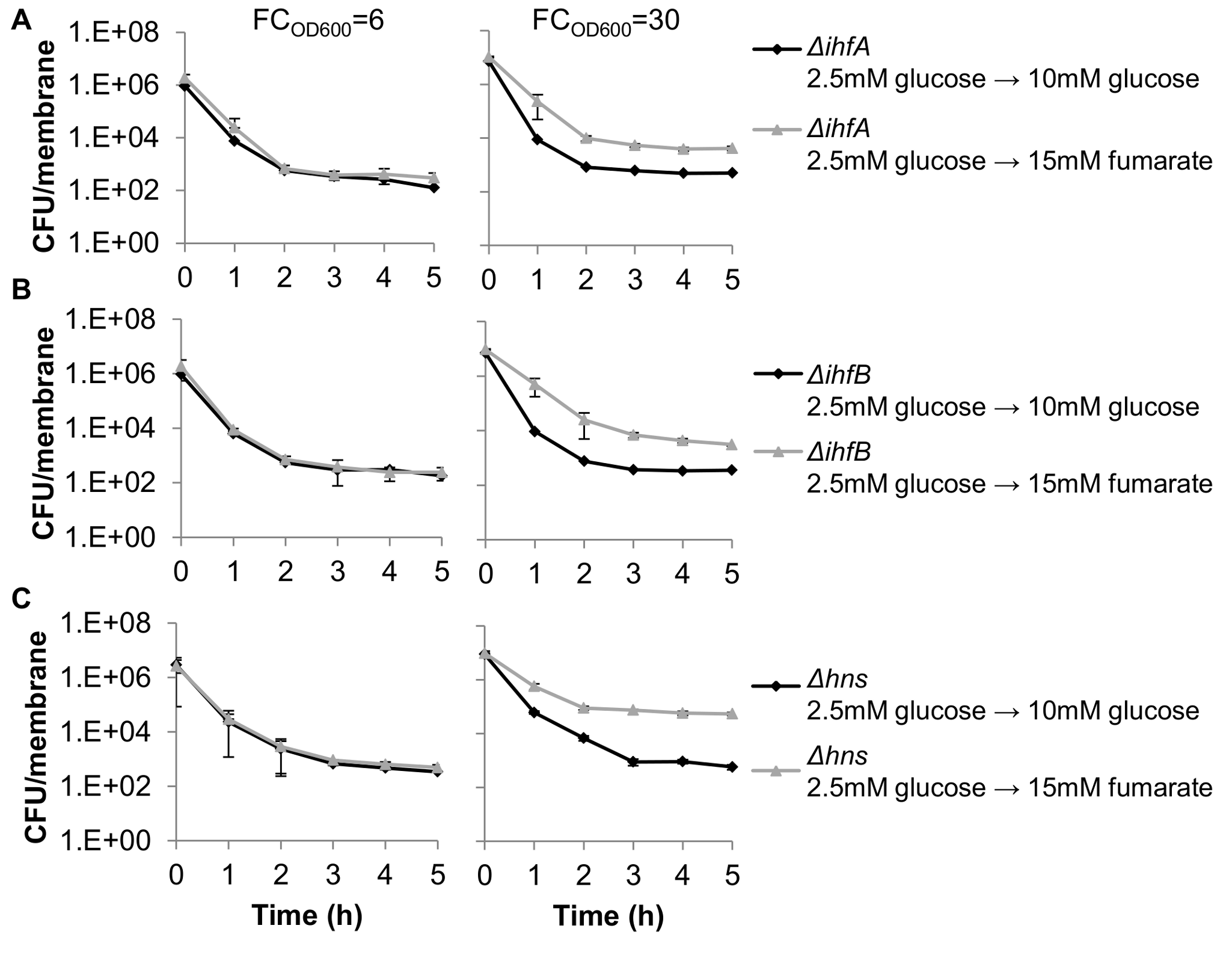

Supplement: Figure S5 — IHF and HNS are not involved in persister formation from carbon source transitions in biofilms. Cells were challenged with 200 μL of 10 μg/mL ofloxacin at FCOD600 = 6 and FCOD600 = 30, representing growth on glucose and growth after glucose exhaustion, respectively (except for glucose-only sample). (A) ΔihfA, (B) ΔihfB, and (C) Δhns produced fold-change increases in persisters (glucose-fumarate persisters/glucose-only persisters) that were not significantly reduced compared to wild-type. Data are averages of 3 independent experiments, error bars indicate standard deviation, and significance was assessed using the null hypothesis that the mutant mean fold-change in persisters was equal to the wild-type fold-change in persisters. (TIF) [file pone.0093110.s005.tif]

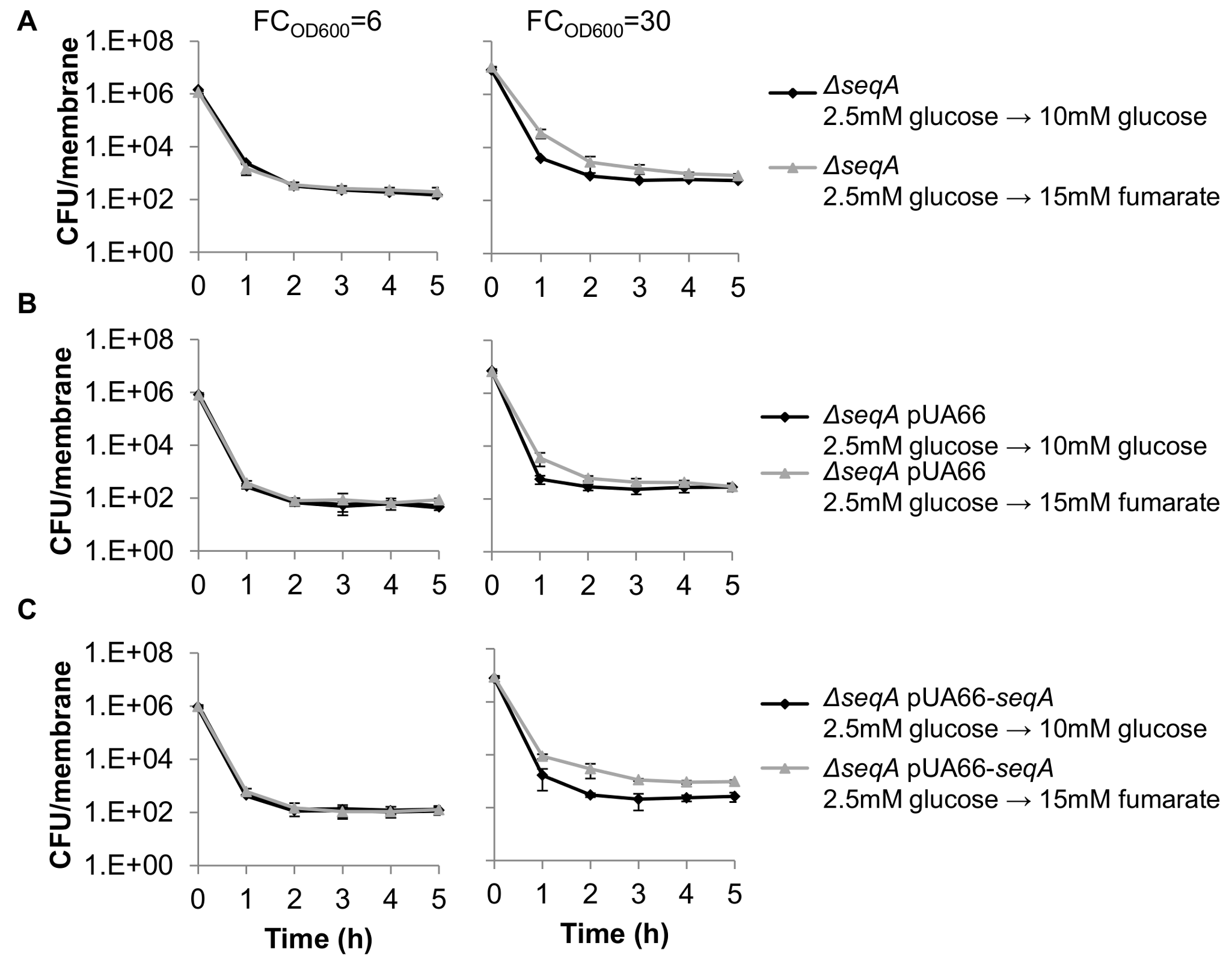

Supplement: Figure S6 — Complementation of FIS and HU. Cells were challenged with 200 μL of 10 μg/mL ofloxacin at FCOD600 = 6 and FCOD600 = 30, representing growth on glucose and growth after glucose exhaustion, respectively (except for glucose-only sample). (A) Δfis pUA66 eliminated persister formation, while (B) Δfis pUA66-dusB-fis restored persister formation. Analogous results were obtained for (C) ΔhupA pUA66 compared to (D) ΔhupA pUA66-hupA and for (E) ΔhupB pUA66 compared to (F) ΔhupB pUA66-hupB. Data are averages of 3 independent experiments, error bars indicate standard deviation, and significance was assessed using the null hypothesis that the mean fold-change in persisters for the complemented strain was equal to the mean fold-change in persisters for the deletion strain carrying the pUA66 vector. (TIF) [file pone.0093110.s006.tif]

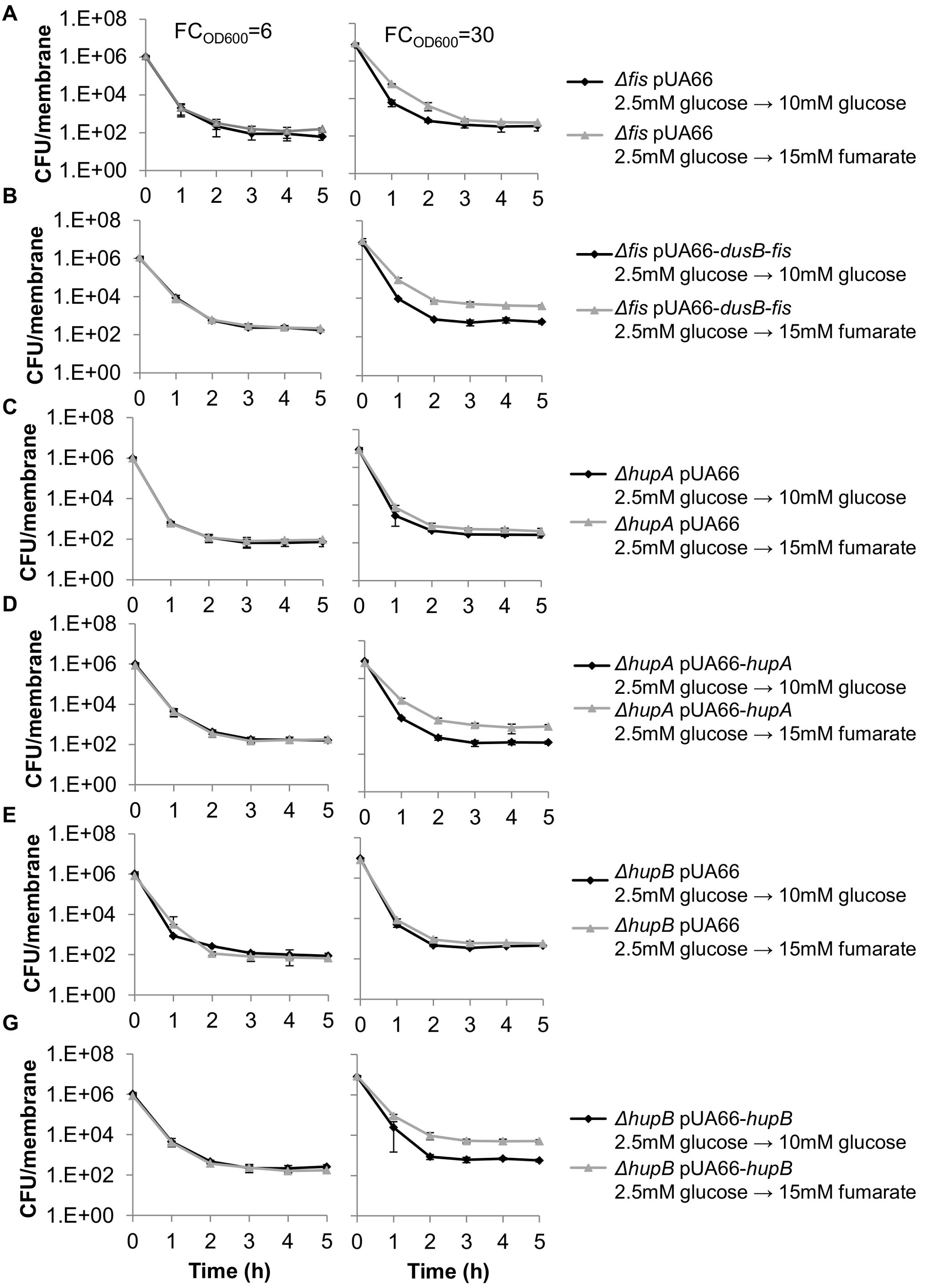

Supplement: Figure S7 — Involvement of SeqA in persister formation from carbon source transitions. Cells were challenged with 200 μL of 10 μg/mL ofloxacin at FCOD600 = 6 and FCOD600 = 30, representing growth on glucose and growth after glucose exhaustion, respectively (except for glucose-only sample). (A) ΔseqA eliminated persister formation compared to wild-type (p<0.05). (B) ΔseqA pUA66 also eliminated persister formation compared to the wild-type, but complementation of ΔseqA with (C) ΔseqA pUA66-seqA did not give a statistically significant increase in persisters compared to the ΔseqA pUA66 control. Data are averages of 3 independent experiments, error bars indicate standard deviation, and significance was assessed using the null hypothesis that the mean fold-change in persisters for the complemented strain was equal to the mean fold-change in persisters for the deletion strain carrying the pUA66 vector. (TIF) [file pone.0093110.s007.tif]

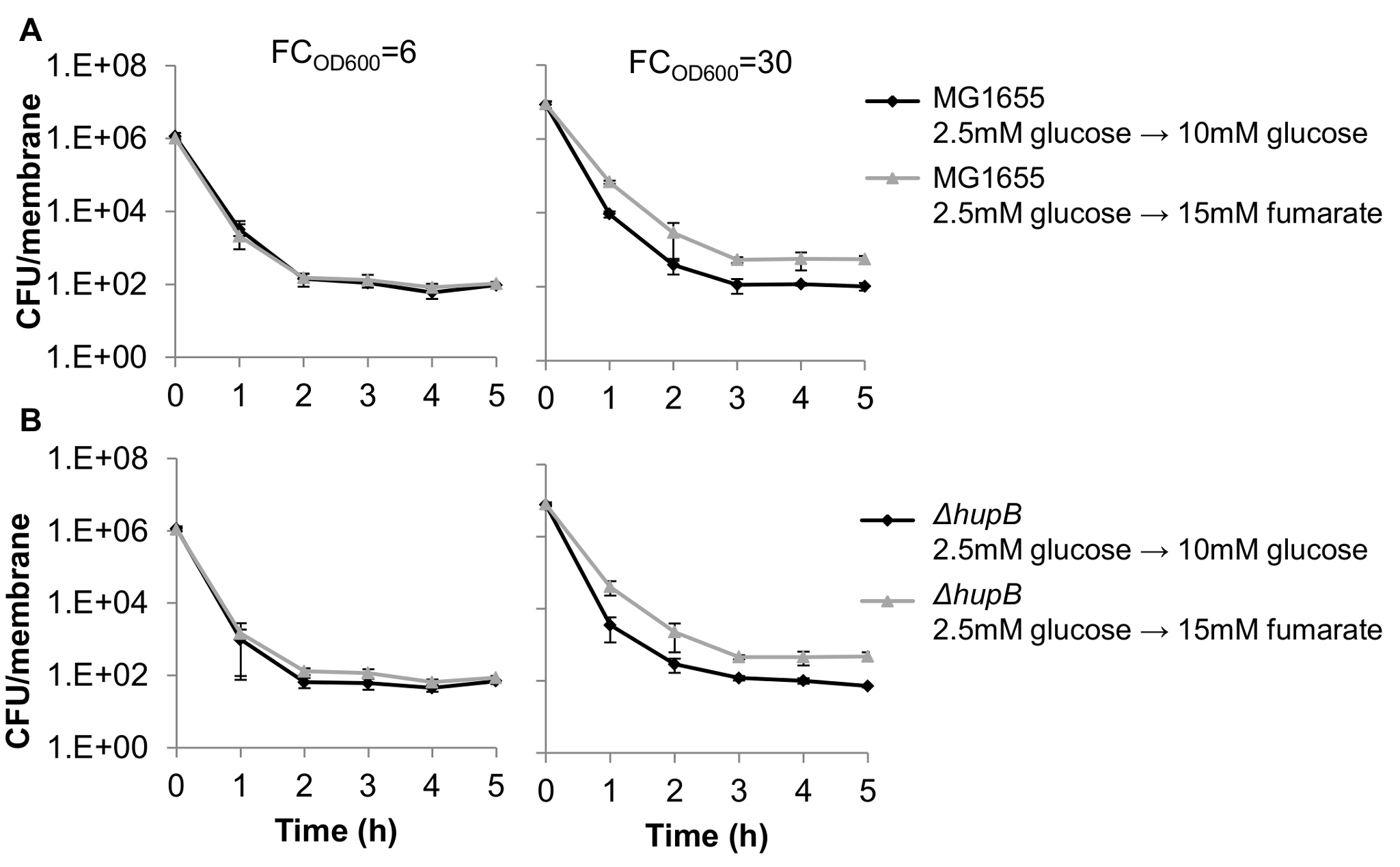

Supplement: Figure S8 — Ampicillin persister formation from carbon source transitions does not depend on HU. Cells were challenged with 200 μL of 750 μg/mL ampicillin at FCOD600 = 6 and FCOD600 = 30, representing growth on glucose and growth after glucose exhaustion, respectively (except for glucose-only sample). (A) Wild-type and (B) ΔhupB resulted in statistically significant 5.4-fold and 6.7-fold increase in persisters, respectively. Data are averages of 3 independent experiments, error bars indicate standard deviation, and significance was assessed using the null hypothesis that the mean CFU levels in two sample sets were equal. (TIF) [file pone.0093110.s008.tif]

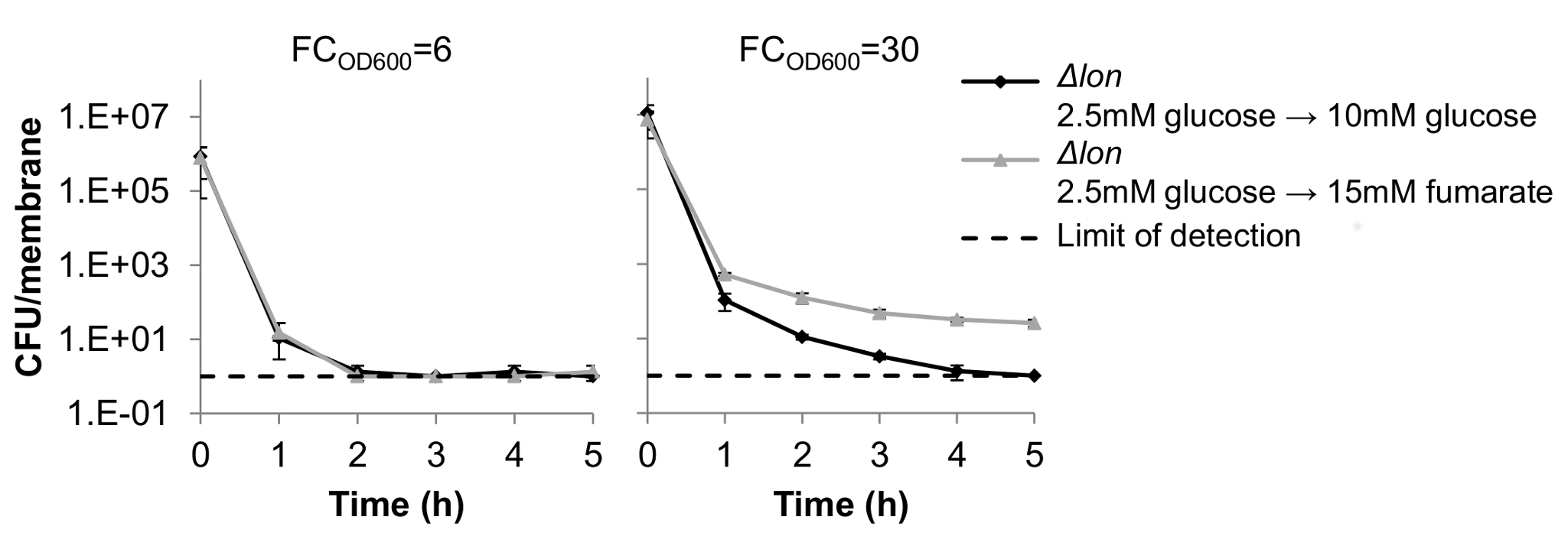

Supplement: Figure S9 — Involvement of Lon in persister formation from carbon source transitions in biofilms. Cells were challenged with 200 μL of 10 μg/mL ofloxacin at FCOD600 = 6 and FCOD600 = 30, representing growth on glucose and growth after glucose exhaustion, respectively (except for glucose-only sample). Δlon did not eliminate persister formation due to a carbon source transition in biofilms. The limit of detection was 1 CFU/membrane. Data are averages of 3 independent experiments and error bars indicate standard deviation. (TIF) [file pone.0093110.s009.tif]
