# Supplementary material for: Nutrient Transitions Are a Source of Persisters in Escherichia coli Biofilms
Source: PLoS One. 2014 Mar 25;9(3):e93110. doi: 10.1371/journal.pone.0093110 (PMC3965526; doi:10.1371/journal.pone.0093110)
Supplement: Table S1 — Glucose concentrations (nmol/membrane). Glucose concentration measurements were taken at each FCOD600 = 6 and = 30 for all strains in both 10 mM glucose and 15 mM fumarate samples. Measurements were made using an Amplex Red Glucose/Glucose Oxidase Kit (Invitrogen). Three replicates were conducted for each mutant and condition and error indicates standard deviation. (DOC) [file pone.0093110.s010.doc]

**Table S1. Glucose concentrations (nmol/membrane)**

|  | 10mM glucose | | 15mM fumarate | |
| --- | --- | --- | --- | --- |
| Bacterial Strain | FC_OD600_=6 | FC_OD600_=30 | FC_OD600_=6 | FC_OD600_=30 |
| wild-type | 62.5 ± 0.61 | 64.1 ± 1.59 | 36.2 ± 9.60 | 3.1 ± 1.54 |
| *Δfis* | 50.6 ± 26.41 | 66.7 ± 3.30 | 30.2 ± 33.97 | 0 ± 0 |
| *Δhns* | 62.3 ± 2.20 | 61.1 ± 4.16 | 15.4 ± 6.72 | 4.5 ± 1.48 |
| *ΔhupA* | 65.2 ± 1.76 | 64.3 ± 3.56 | 18.2 ± 7.31 | 0 ± 0 |
| *ΔhupB* | 62.6 ± 3.85 | 62.2 ± 1.38 | 17.7 ± 1.99 | 0 ± 0 |
| *ΔihfA* | 69.6 ± 2.27 | 70.3 ± 2.95 | 17.1 ± 5.03 | 2.8 ± 4.32 |
| *ΔihfB* | 71.8 ± 2.57 | 73.7 ± 4.22 | 12.3 ± 9.25 | 2.3 ± 2.29 |
| *ΔrelA* | 61.4 ± 2.76 | 61.2 ± 2.42 | 11.1 ± 1.19 | 1.1 ± 0.92 |
| *ΔseqA* | 71.6 ± 1.98 | 70.0 ± 4.40 | 15.1 ± 3.80 | 0 ± 0 |
