# Supplementary material for: Nutrient Transitions Are a Source of Persisters in Escherichia coli Biofilms
Source: PLoS One. 2014 Mar 25;9(3):e93110. doi: 10.1371/journal.pone.0093110 (PMC3965526; doi:10.1371/journal.pone.0093110)
Supplement: Table S2 — Raw values for Figure 2A . FCOD600 prior to antibiotic treatment and persister measurements after 5 hours of treatment with 200 μL of 10 μg/mL ofloxacin were taken at the specified time points for a panel of secondary carbon sources using glucose as a primary carbon source. These data were used to linearly interpolate values to generate the heat map for Figure 2A. (DOC) [file pone.0093110.s011.doc]

Table S2. Raw values for Figure 2A

| Time (h) | 10mM glucose | 10mM  fructose | 10mM gluconate | 20mM glycerol | 5mM lactose | 15mM fumarate | 15mM succinate |
| --- | --- | --- | --- | --- | --- | --- | --- |
|  | Persisters (CFU/membrane) | | | | | | |
| 3 | 217 ± 36 | 220 ± 80 | 247 ± 31 | 273 ± 127 | 200 ± 20 | 273 ± 129 | 267 ± 31 |
| 4 | 390 ± 137 | 367 ± 12 | 340 ± 100 | 493 ± 94 | 440 ± 197 | 507 ± 101 | 387 ± 31 |
| 5 | 557 ± 194 | 753 ± 31 | 607 ± 261 | 3073 ± 1150 | 1467 ± 1175 | 2593 ± 1124 | 893 ± 185 |
| 6 | 813 ± 163 | 873 ± 83 | 867 ± 81 | 5733 ± 240 | 3400 ± 721 | 7733 ± 2157 | 5267 ± 1501 |
| 7 | 1733 ± 315 | 1313 ± 343 | 1987 ± 751 | 8667 ± 1179 | 4667 ± 924 | 8667 ± 416 | 8400 ± 1249 |
| 8 | 5467 ± 622 | 5467 ± 416 | 5600 ± 346 | 8733 ± 467 | 4667 ± 1172 | 14933 ± 9751 | 9400 ± 2253 |
|  | FC_OD600_ | | | | | | |
| 3 | 4.67 ± 1.03 | 4.67 ± 1.15 | 6 ± 2.00 | 4.67 ± 1.15 | 4 ± 0.00 | 5.33 ± 1.15 | 4.67 ± 1.15 |
| 4 | 9.33 ± 2.07 | 11.33 ± 2.31 | 9.33 ± 1.15 | 11.33 ± 1.15 | 8.67 ± 1.15 | 12 ± 2.00 | 9.33 ± 2.31 |
| 5 | 20.67 ± 2.73 | 18 ± 3.46 | 18.67 ± 4.16 | 14.67 ± 3.06 | 13.33 ± 5.03 | 16 ± 2.00 | 16 ± 2.00 |
| 6 | 39 ± 1.10 | 28.67 ± 6.11 | 35.33 ± 11.01 | 23.33 ± 1.15 | 25.33 ± 9.45 | 27.33 ± 3.06 | 24.67 ± 7.02 |
| 7 | 77.67 ± 5.29 | 54 ± 4.00 | 64 ± 17.09 | 36 ± 2.00 | 42 ± 9.17 | 39.33 ± 2.31 | 36.67 ± 5.77 |
| 8 | 155.67 ± 22.46 | 92 ± 10.58 | 112.67 ± 36.07 | 57.33 ± 7.58 | 74 ± 32.18 | 62.67 ± 8.33 | 60 ± 12.49 |
