# Supplementary material for: Nutrient Transitions Are a Source of Persisters in Escherichia coli Biofilms
Source: PLoS One. 2014 Mar 25;9(3):e93110. doi: 10.1371/journal.pone.0093110 (PMC3965526; doi:10.1371/journal.pone.0093110)
Supplement: Table S3 — Bacterial strains and plasmids. (DOC) [file pone.0093110.s012.doc]

Table S3. Bacterial strains and plasmids

| Strain | Genotype | Source or Reference |
| --- | --- | --- |
| MG1655 | F- λ- ilvG- rfb-50 rph-1 | ATCC 700926 |
| SA008 | MG1655 Δ*fis*::kan | P1 Keio mutant x MG1655 |
| SA012 | MG1655 *Δhns*::kan | P1 Keio mutant x MG1655 |
| SA014 | MG1655 Δ*hupA*::kan | P1 Keio mutant x MG1655 |
| SA015 | MG1655 Δ*hupB*::kan | P1 Keio mutant x MG1655 |
| SA016 | MG1655 Δ*ihfA*::kan | P1 Keio mutant x MG1655 |
| SA017 | MG1655 Δ*ihfB*::kan |  |
| SA021 | MG1655 Δ*relA*::kan | P1 Keio mutant x MG1655 |
| SA026 | MG1655 Δ*seqA*::kan | P1 Keio mutant x MG1655 |
| SA034 | MG1655 *lacI^q^* P_T5_* *gfp ΔlacZYA* Kan^r^ | This work |
| SA035 | MG1655 Δ*relA* | SA021 cured of Kan^r^ |
| SA036 | MG1655 Δ*fis* | SA008 cured of Kan^r^ |
| SA037 | MG1655 Δ*hupA* | SA014 cured of Kan^r^ |
| SA038 | MG1655 Δ*hupB* | SA015 cured of Kan^r^ |
| SA039 | MG1655 Δ*seqA* | SA026 cured of Kan^r^ |
| SA040 | MG1655 Δ*lon*::kan | P1 Keio mutant x MG1655 |
| Plasmid | Genotype | Source or Reference |
| pET11-a | Vector, pBR322 ori, *bla,* T7 expression | Novagen |
| pUA66 | Vector, SC101ori, Kan^r^, *gfp*mut2 reporter |  |
| pSA03 | pUA66 P*_malK_*-*gfp*mut2 Kan^r^ |  |
| pSA10 | pUA66 P*_relA_*-*relA* Kan^r^ | This work |
| pSA11 | pUA66 P*_hupA_*-*hupA* Kan^r^ | This work |
| pSA12 | pUA66 P*_hupB_*-*hupB* Kan^r^ | This work |
| pSA13 | pUA66 P*_dusB_-dusB-fis* Kan^r^ | This work |
| pSA14 | pUA66 P*_seqA_-seqA* Kan^r^ | This work |

* T5 promoter (P_T5_) used in this study is a strong, IPTG inducible promoter under the control of two lac operator sites.
