# Supplementary material for: Nutrient Transitions Are a Source of Persisters in Escherichia coli Biofilms
Source: PLoS One. 2014 Mar 25;9(3):e93110. doi: 10.1371/journal.pone.0093110 (PMC3965526; doi:10.1371/journal.pone.0093110)
Supplement: Table S4 — DNA primers for plasmid construction. (DOC) [file pone.0093110.s013.doc]

**Table S4**. DNA primers for plasmid construction

| Plasmid | Forward | Reverse |
| --- | --- | --- |
| pSA10 | 5'-GCGCGGCTCGAGTAGCCATCTTGGTCATGTTGAACTGGTA-3' | 5'-GCGCGGCCTGCAGGGCCTAACTCCCGTGCAACC-3' |
| pSA11 | 5'-GCGCGGCTCGAGTAGCGATGCTGGAAATGACTCAGG-3' | 5'-GCGCGGCCTGCAGGCTGTTCACTGCCACGCAATC-3' |
| pSA12 | 5'-GCGCGGCTCGAGTAGCACCGTCTGGTATGCAGGTT-3' | 5'-GCGCGGCCTGCAGGTTAGTTTACCGCGTCTTTCAGT-3' |
| pSA13 | 5’-GCGCGGCTCGAGTAGCACCGGTCGTAAGAATTAACCTTG-3’ | 5'-GCGCGGCCTGCAGGAACCTGAATTAGTTCATGCCGTAT-3' |
| pSA14 | 5'-GCGCGGCTCGAGTAGCAGCCAGGCTTGACGCTATC-3' | 5'-GCGCGGCCTGCAGGTTAGATAGTTCCGCAAACCTTC-3' |
