# Supplementary material for: Nutrient Transitions Are a Source of Persisters in Escherichia coli Biofilms
Source: PLoS One. 2014 Mar 25;9(3):e93110. doi: 10.1371/journal.pone.0093110 (PMC3965526; doi:10.1371/journal.pone.0093110)
Supplement: Table S5 — DNA primers for qPCR. (DOC) [file pone.0093110.s014.doc]

**Table S5**. DNA primers for qPCR

| Gene | Forward | Reverse |
| --- | --- | --- |
| *rrsA* (16S) | 5’-GAAGAAGCACCGGCTAACTC-3’ | 5’-TGCCAGTATCAGATGCAGTTC-3’ |
| *rrlA*  (23S) | 5’-TTCCTTGTCGGGTAAGTTCC-3’ | 5’-AGCCTCCCACCTATCCTACA-3’ |
| *phzM* | 5’-CGGCGAAGACTTCTACAGCTA-3’ | 5’-CAGGATGGCCTTGGTCAAT-3’ |
