# Supplementary material for: Nutrient Transitions Are a Source of Persisters in Escherichia coli Biofilms
Source: PLoS One. 2014 Mar 25;9(3):e93110. doi: 10.1371/journal.pone.0093110 (PMC3965526; doi:10.1371/journal.pone.0093110)
Supplement: Materials and Methods S1 — (DOCX) [file pone.0093110.s015.docx]

**Supporting Information**

**Supporting Material and Methods**

*Plasmid constructions*

For complementation, plasmids pSA10, pSA11, pSA12, pSA13, and pSA14 were constructed by cloning the *relA*, *hupA*, *hupB*, *dusB-fis*, and *seqA* loci, respectively, with the designated promoter and transcriptional regulation, into the XhoI/SbfI sites of the pUA66 vector using primers specified in Table S4. These plasmids were transformed into the appropriate genetic deletion background without the kanamycin resistance cassette for complementation after strains were cured of the kanamycin resistance cassette using FLP recombinase.

*Synthesis of control RNA*

The *phzM* gene of *Pseudomonas aeruginosa* (PAO1) was used as a control gene for qPCR. The *phzM* gene was amplified from purified *P. aeruginosa* genomic DNA with NdeI and BamHI sites incorporated into the primers to include restriction sites before and after the coding sequence. The amplification primers used were GCGCATCATATGAATAATTCGAATCTTGCTG (forward) and GCGCGGGGATCCTCAGGCCCTGGCAGC (reverse). The *phzM* amplicon was restriction digested and inserted into the pET11-a vector at the NdeI and BamHI sites. This vector possesses the T7 promoter upstream of the insertion site. The pET11-a-*phzM* plasmid was linearized by digesting with HindIII. The *phzM* RNA was synthesized *in vitro* from the linearized pET11-a-*phzM* plasmid using the T7 High Yield RNA Synthesis Kit (New England Biolabs). The RNA product was purified using the RNA cleanup protocol from the RNeasy Mini Kit following the manufacturer’s protocol (Qiagen). Total RNA concentration and purity was analyzed by measuring optical density at 260nm and 280nm using NanoDrop 1000 (Thermo Scientific).

*qPCR analysis*

Wild-type and *ΔrelA* were prepared as described in the Materials and Methods and grown on 15mM fumarate agar. At FC_OD600_=14 during the growth arrest period, membranes were aseptically removed from the agar and vortexed in 2mL of PBS. 1.5mL of the bacterial suspension was centrifuged, 1.3mL of the supernatant was removed to concentrate cells, and 400µL RNAprotect bacterial reagent (Qiagen) was immediately added according to manufacturer’s protocol. Pellets were stored at -80°C until purification. Pellets were thawed to room temperature and 10ng of the control *phzM* RNA was spiked into the sample as control for RNA purification and a normalization product for qPCR. RNA extraction was performed with the RNeasy mini (Qiagen) according to manufacturer’s protocol. Samples were treated with RNase-free DNase I (Qiagen) on-column treatment during purification. Total RNA concentration and purity was estimated by measuring optical density at 260nm and 280nm using NanoDrop 1000 (Thermo Scientific).

cDNA was generated using ~15-30ng of purified RNA in a total volume of 10µL with random hexamer primers according to the TaqMan® Reverse Transcription (Life Technologies) protocol. Real-time qPCR was performed using an ABI Prism 7900H Sequence Detection System (Applied Biosystems) with 40 amplification cycles using SYBR Green PCR Master Mix as signal reporter. Each reaction composed of 1µL of cDNA from RT reaction, 250 nM sense and antisense primers (Table S5) in a total volume of 20 μL. qPCR was done in a 96-well microtiter PCR plate. Each sample consisted of three biological replicates each with two technical replicates. To assess for reagent and genomic DNA contamination, no template and no reverse transcriptase controls were included. The gene expression in each strain was determined using absolute quantification through a standard curve that related known concentrations to the corresponding threshold cycle of either genomic DNA for the rRNA genes or *phzM* cDNA for the control RNA. All standard curves possessed an R^2^ value ≥ 0.99. rRNA transcripts were normalized to the number of *phzM* transcripts present in the sample. Fold-change in expression for *ΔrelA* compared to wild-type was calculated for samples run in parallel with the same extraction, purification, cDNA synthesis, and qPCR amplification. Statistical significance was assessed using a t-test where the null hypothesis states that the fold-change of Δ*relA* transcripts to wild-type transcripts have a mean of 1 against the alternative that the mean is not 1 where a p-value < 0.05 rejects the null hypothesis.

*Confirmation of Lon protease deletion*

*Δlon* was confirmed using PCR and two sets of primers. One set confirmed the presence of the kanamycin resistance cassette in the proper chromosomal location using a forward primer upstream of *lon*, 5’-GATTTATGGCAAGCCGGAAG-3’, and a kanamycin resistance cassette reverse primer, 5’- ATGATGGATACTTTCTCGGCAGGAG-3’. The second set confirmed the absence of a duplicate gene, and contained primers within the gene of interest with an internal *lon* forward, 5'-GCGTATTTCTGCGCTCTCTG-3', and an internal *lon* reverse, 5'-TAGTGGTCGCTGAACGCTAC-3'.

*Persistence measurements for Δlon*

Persister enumeration for the *Δlon* strain was carried out as described in the main text with the following modification. Following antibiotic treatment at designated time points, membranes were aseptically removed from the agar, vortexed in 1mL of PBS, washed and serially diluted in PBS, and the entire sample was spotted onto LB agar to achieve a limit of detection of 1 CFU/membrane.

**Supporting References**

1. Kohanski MA, Dwyer DJ, Hayete B, Lawrence CA, Collins JJ (2007) A common mechanism of cellular death induced by bactericidal antibiotics. Cell 130: 797-810.

2. Baba T, Ara T, Hasegawa M, Takai Y, Okumura Y, et al. (2006) Construction of Escherichia coli K-12 in-frame, single-gene knockout mutants: the Keio collection. Mol Syst Biol 2: 2006 0008.

3. Amato Stephanie M, Orman Mehmet A, Brynildsen Mark P (2013) Metabolic Control of Persister Formation in Escherichia coli. Mol Cell 50: 475-487.

4. Datsenko KA, Wanner BL (2000) One-step inactivation of chromosomal genes in Escherichia coli K-12 using PCR products. Proc Natl Acad Sci U S A 97: 6640-6645.

5. Zaslaver A, Bren A, Ronen M, Itzkovitz S, Kikoin I, et al. (2006) A comprehensive library of fluorescent transcriptional reporters for Escherichia coli. Nat Methods 3: 623-628.

6. Bujard H, Gentz R, Lanzer M, Stueber D, Mueller M, et al. (1987) A T5 promoter-based transcription-translation system for the analysis of proteins in vitro and in vivo. Methods Enzymol 155: 416-433.
